# Trust behavior in Parkinson’s disease: results of a trust game experiment

**DOI:** 10.1186/s12883-015-0374-5

**Published:** 2015-07-31

**Authors:** Andrija Javor, René Riedl, Matthias Kirchmayr, Mirella Reichenberger, Gerhard Ransmayr

**Affiliations:** Department of Neurology and Psychiatry, Linz General Hospital, Faculty of Medicine, Johannes Kepler University Linz, Krankenhausstrasse 9, 4021 Linz, Austria; School of Management, University of Applied Sciences Upper Austria, Wehrgrabengasse 1-3, 4400 Steyr, Austria; Department of Business Informatics – Information Engineering, Johannes Kepler University Linz, Altenberger Strasse 69, 4040 Linz, Austria

**Keywords:** Parkinson, Trust game, Risk, Behavior, Non-motor symptoms, Game-of-dice task, Behavioral neurology

## Abstract

**Background:**

Parkinson’s Disease (PD) is a neurodegenerative disease characterized by motor symptoms, but in which behavioral and cognitive disturbances are also common. Trust, due to its pervasiveness in society, has become a major research topic in several scientific disciplines. However, empirical evidence for trust behavior in neurological patients, and specifically for movement disorders such as PD, is missing. Evidence from healthy subjects, however, indicates that three brain regions are involved in trust perceptions and behavior, namely the limbic system, basal ganglia, and frontal cortex. PD affects all these brain regions. Therefore, we hypothesized that PD patients and healthy controls show differences in trust behavior.

**Methods:**

We conducted an experiment using the trust game, an established paradigm to investigate trust behavior in both patient and healthy populations alike, controlling for risky decision making. Twenty patients suffering from PD diagnosed according to UK PDS Brain Bank criteria and twenty healthy controls (matched for age, gender, education, and income) were recruited. We excluded those suffering from clinically relevant neuropsychiatric comorbidities.

**Results:**

We found that PD patients exhibit significantly lower levels of trust than do healthy controls. Importantly, our results cannot be explained by lower levels of risk-taking. Moreover, our results indicate that the trust deficit is independent of medication, disease duration, and severity of motor symptoms.

**Conclusion:**

Application of a standard procedure for measuring trust behavior revealed that PD patients exhibit lower levels of trust in other humans than do healthy controls. Against this background we make a call for further research to determine the underlying pathophysiology of reduced trust in PD.

## Background

Parkinson’s Disease (PD) is a chronic and progressive neurodegenerative disease characterized by the motor symptoms bradykinesia, rigidity, resting tremor, and impaired postural reflexes [1], but in which non-motor symptoms including behavioral and cognitive disturbances are also common [2].

Trust is a fundamental prerequisite for various kinds of relationships in both private and public life, is essential for the functioning of society in general [3, 4], and has therefore recently become a major research topic in various scientific disciplines, including psychology (e.g. [5]), economics (e.g. [3]), information systems research (e.g. [6]), social neuroscience (e.g. [7]), and psychiatry (e.g. [8]). Nevertheless, empirical evidence for trust behavior in neurological patients, and specifically for movement disorders such as PD, is still missing. This is problematic given the importance of trust in the patient-physician relationship and the impact of trust, among many other variables, on therapy adherence [9], which is low in PD [10].

Even though several definitions of trust exist, most concepts conceptualize trust as a belief, expectation, attitude, or behavior. Generally, trust may be defined as a psychological state comprising the intention to accept vulnerability based on positive expectations about the actions of another party (the trustee) [11, 12]. Formally, trust is therefore the subjective assessment of the probability that someone will behave in a trustworthy manner [13]. Other psychological states are preconditions for trust, particularly risk perception, substantially regulated in the amygdala, insular cortex and other parts of the limbic system [14], because without perceived risk no trust is needed.

The goal of trusting another individual is to realize a reward (e.g., through beneficial cooperation), a fact that explains the importance of the basal ganglia, particularly the striatum, and the reward system in general, for trust behavior [15]. Moreover, positive expectations with regard to the future behavior of the trustee are important in trust situations, and such expectations are often derived through the process of mentalizing (i.e., inferring other actors’ intentions), which is related to frontal lobe function (particularly activation in the paracingulate and the medial prefrontal cortex) [16]. For an interdisciplinary discussion of trust, see [17]. In line with these conceptual relationships between trust and risk perception, reward, and mentalizing, many studies in the neurobiological stream of research have found the corresponding three brain regions and their reciprocal connections to be activated in trust situations, namely the limbic system, basal ganglia, and the frontal cortex [12, 15, 18, 19].

It is a well-established fact that PD affects several brain areas, many of which are related to trust. PD pathologically transforms the limbic system [20], even in non-demented and non-depressed patients [21], and leads to pathological functioning of striatal circuits [22]. Additionally, dopaminergic medication has significant impact on risk perception and decision making in PD patients shifting decisions to higher risk choices (e.g. [23, 24]). Frontal executive functions are long known to be affected in PD (for a recent review, see [25]), and disturbances in mentalizing abilities have also been reported (for a review, see [26]).

Because of these impairments we hypothesized that a difference in trust behavior exists between PD patients and healthy controls. However, current evidence did not permit us to anticipate the directionality of this hypothesized effect. On the one hand, as a result of PD patients’ deficiencies in brain regions associated with trust (limbic system, basal ganglia, frontal cortex), such patients could be expected to exhibit lower levels of trust behavior than healthy controls. On the other hand, as a result of the known positive correlation between dopamine and trust (for a review, see [12]) and the fact that we planned to test patients taking anti-Parkinson medication in order to simulate real-life clinical settings, patients’ trust behavior might be elevated under treatment.

Against this background, we formulated the following hypothesis, which we tested in a laboratory experiment: *Trust behavior of PD patients differs from that of healthy controls.*

## Methods

### Participants

All patients suffering from PD who fulfilled strict diagnostic criteria (UK PDS Brain Bank [27]), were right-handed, Caucasian, and had no psychiatric comorbidities in their history were considered for enrollment in the order of presentation at the Movement Disorders Outpatient Clinic. In an opt-out approach, patients were contacted without their volunteering to take part in the research and were excluded only if they declared their unwillingness to participate. Even though patients had no history of psychiatric comorbidities, we screened for and in a second step excluded those patients suffering from anxiety, depression, dementia, impulsive-compulsive disorders and/or apathy at the time of the experiment in order to minimize possible effects of these disorders on trust behavior. As a screening tool, we used the complete version of the Patient Health Questionnaire (PHQ-D [28]) and the Questionnaire for Impulsive-Compulsive Disorders (QUIP [29]); excluded were patients who met pathological criteria. We screened for dementia according to the Diagnostic and Statistical Manual of Mental Disorders (DSM IV [30]) using the Mini Mental State Examination (MMSE [31]) and excluded those with an MMSE < 26. Finally, we applied the Lille Apathy Rating Scale (LARS [32]) to exclude those suffering from apathy (cut-off point > −15).

Twenty patients ultimately participated in the study (mean age 72.35 years, SD 9.16, equally gendered). To better characterize our patient sample we used the Unified Parkinson's Disease Rating Scale III (UPDRS [33]) and the Hoehn & Yahr Scale [34] to evaluate the severity of motor symptoms and recorded anti-Parkinson, as well as other ongoing medication, disease duration, age, education, income, and religion using the corresponding, slightly adjusted National Institute of Neurological Disorders and Stroke Common Data Elements [35].

All patients were mildly to moderately ill (mean UPDRS III score under treatment 19.85, SD 10.59; median Hoehn & Yahr Stage 2) and received L-DOPA treatment (mean daily dose 337.5 mg, SD 303). Nine patients were on supplementary pramipexole medication (mean daily dose 1.85 mg, SD 1.1), four patients took ropinirole (mean daily dose 2.5 mg, SD 1.0), and two rotigotine (mean daily dose 6 mg, SD 2.83). The mean total L-DOPA equivalent daily dose (L-DOPA + dopamine agonists) was 448.782 mg (SD 371.443) and was calculated according to [36]. Four patients reported mild predictable motor fluctuations, three of whom also mentioned rare dyskinesias (1 %-25 % of the day). All but three patients had a Schwab and England Activities of Daily Living Scale score [37] of 80 % or more.

As control subjects we recruited twenty right-handed, Caucasians (mean age 68.4 years, SD 10.0, equally gendered) with no history of neurological or psychiatric disease, with a normal neurological status, and who passed the same exclusion criteria on MMSE and PHQ-D as the patient group and were therefore considered healthy. The controls were usually recruited from among the patients’ spouses or partners to minimize differences in age, income, education, or religion, as these factors might affect trust behavior [38]. Table 1 summarizes the characteristics of the patient and control groups.

**Table 1:** Characteristics of the patient and control groups

Variable	Group	
	Patients, *n* = 20 mean ± SD	Controls, *n* = 20 mean ± SD
Female gender (%)	50	50
Age	72.35 ± 9.16	68.4 ± 10
Income class^1^ (median)	3	4
Years of education^2^	10.9 ± 4.03	11.3 ± 5.11
UPDRS III	19.85 ± 10.59	-
Hoehn & Yahr Stage (median)	2	-
MMSE	28.4 ± 1.6	28.95 ± 0.99
LEDD (mg/day)	448,782 ± 371,443	-
Disease duration (months)	39.21 ± 41.82	-

See Endnotes for further information on income and education variables

*UPDRS-III* Unified Parkinson’s Disease Rating Scale Part III, *MMSE* Mini Mental State Examination, *LEDD* L-DOPA equivalent daily dose of dopamine agonists + L-DOPA dose

### Experimental procedure

Recently, Javor et al. [39] argued that economic games constitute an appropriate experimental paradigm for studying behavioral symptoms in neurological diseases. First, the rules of the game can be understood easily. Second, the game offers the possibility of quantifying differences in *actual behavior* between patients and healthy controls, and hence differs from patient or care giver-based questionnaires with which antecedents of trust behavior, such as beliefs or attitudes, can be measured.

One specific economic game, the trust game [40] (for details, see below), is the most frequently used means of measuring trust in healthy subjects and patients alike, and has been shown to reliably distinguish trust from other constructs such as altruism [41]. Using this paradigm, impaired trust was found for several psychiatric patient populations (e.g., psychosis and borderline personality disorder, for a review, see [42]). Importantly, even though we are not aware of a peer-reviewed scientific study that employed the trust game to study the trust behavior of PD patients, it has been shown that non-demented patients suffering from PD are able to understand the rules of the game (see [43], a study on altruistic punishment). Thus, the trust game is an appropriate experimental paradigm for studying trust behavior in PD patients.

The study was approved by the ethics committee of the Federal State Upper Austria. Written consent to participate in the study was obtained from all subjects prior to the experiment. When subjects entered the laboratory for the experiment, they received written instructions explaining the rules and payoff structure for the experiment (the instructions are available from the first author on request). We subsequently checked whether they understood the information by going through several hypothetical examples. All included subjects answered the control questions correctly.

Each round of our version of the trust game involves two players (the trustor and the trustee). The trustor plays the first move, the trustee the second move of the game. This study focused on the trustor, whose role was played by the participants on a computer. The initial endowment made to the participants was €10. Subjects were told they would be playing with real money and that at the end of the experiment they would receive the mean payoff across all rounds of the game. Thus, the goal of each round and the entire game was to maximize the player's payoff. The participants were further told that they would be playing against human beings, but actually played against a randomized computer-generated strategy that simulated a trustee’s behavior. The trustee role was illustrated with human face images from an established face database [44, 45] that were presented on a computer screen. We selected 16 faces with an emotionally neutral expression in order to exclude effects of a possible impairment of emotion recognition in PD patients [46]. All faces had a consistent age range (between 20 and 30 years) and were equally gendered.

The participants could decide to send any amount of their initial endowment to the trustee, but had to use whole numbers between €0 and €10. This decision was made knowing that this amount would be multiplied by 6 by the investigators and given to the trustee, who could then decide whether to send money back to the participant (and, if so, how much). Thus, each subject saw 16 trustee faces, presented in random order, and had to decide how much of the €10 endowment to send to each of the trustees. Randomization of the order of trustees and trustee behavior was implemented to minimize effects between the consecutive rounds of the game. No participant interacted with the same trustee twice (“one-shot trust games”) in order to rule out possible effects of reward-based learning on trust game behavior. We used the monetary amount sent by each participant as a measure of trust behavior.

After playing the trust game participants were explained the rules of the Game-of-Dice Task. Risk-taking is considered a standard control condition in trust research, because humans generally take risks less willingly when the cause of uncertainty is another person, and therefore it is import to distinguish trust from non-social risk taking [47]. We included this specific gambling task because it has been extensively used to study risky decision making in PD patients (e.g. [24, 48–52]). Furthermore, the Game-of-Dice Task offers, in the context of our experimental design, advantages over other decision making paradigms, such as the Iowa Gambling task (IGT), because the IGT includes two mechanisms, namely decisions under ambiguity in the first trials and decisions under risk in the latter trials [53, 54]. This game calls for subjects to maximize their fictive starting capital of 1,000 cents in 18 dice rolls. One virtual dice and a shaker cup are used. In each trial subjects have to guess which number will come up on the next roll. They can choose one of the various single numbers or a combination of two numbers, three numbers, or four numbers. Each choice is associated with specific gains and losses depending on the probability of the occurrence of choice: 1,000 cents gain/loss for the choice of a single number (winning probability 1:6), 500 cents gain/loss for the choice of two numbers (winning probability of 2:6), 200 cents gain/loss for the choice of three numbers (winning probability 3:6), and 100 cents gain/loss for the choice of four numbers (winning probability 4:6). Choices with winning probabilities of 1:6 and 2:6 are considered high-risk and disadvantageous, while 3:6 and 4:6 are low-risk and advantageous. This also implies that there are no “intermediate” risk choices. Thus, the winning probabilities can be easily calculated, and the amount of risk associated with each choice is obvious. In the game the dice is rolled and the particular winning number is presented. Participants receive feedback (gain or loss) for their decision in a visual and acoustic way, and the current capital is shown. The maximum final balance that can be achieved in the game is 19,000 cents if the subject always chooses a single number and is successful on each roll of the dice. The maximum negative balance is −17,000 cents if the subject always chooses a single number and is unsuccessful on each roll (the game description for the Game-of-Dice Task was taken from [55] and slightly modified to improve comprehension). We used the number of risky choices (winning probabilities 1:6 and 2:6) in 18 consecutive rounds of the game as a measure of risk-taking. Actual payment reflecting the participants’ gain took place at the very end of the experiment.

### Statistical analysis

The statistical analysis was performed with SPSS® and R®. Considering our formulated hypothesis, we used two-tailed statistical testing for trust behavior. In analogy, we chose two-tailed statistical testing for risky decision making in the Game-of-Dice Task. Furthermore, we determined the effect sizes of our statistical tests and run a regression analysis to explore the relationship between trust behavior and other variables of our study population.

## Results

The PD patient and control groups were comparable in gender, age, income class, education, and religion (see Table 1), as there was no significant difference in age (mean patients: 72.35, SD 9.16; mean controls 68.4, SD 10.0; Wilcoxon test; *z* = −1.189; *P* = 0.234), income (classified in 7 categories^1^; median patients 4; median controls 3; Wilcoxon test; *z* = −0.771; *P* = 0.440), education^2^ (mean education years patients *y* = 10.9, SD 4.02; controls *y* = 11.3, SD 5.11; Wilcoxon test; *t* = −0.210; *P* = 0.833), or religion (all participants were roman-catholic).

**Fig. 1:**
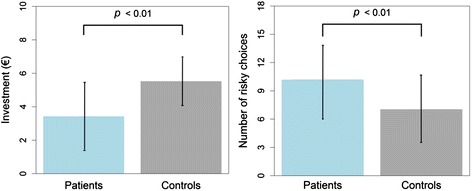
Results of the trust game (left side) and the Game-of-Dice Task (right side). The diagram on the left shows overall mean investments in the trust game (trust behavior). The diagram on the right shows the mean number of risky choices in the Game-of-Dice Task (risky decision making)

Trust behavior differed significantly between the patient and control groups (see Fig. 1). Specifically, the mean investment amount was significantly lower in the PD patient group than in the control group (mean patients: €3.43, SD 2.0; mean controls: €5.53, SD 1.56; Wilcoxon test; *z* = −3.179, P = 0.001; two-sided test, n = 20 in each group), indicating reduced trust in PD. Further calculation revealed an effect size of *r* = 0.71, indicating a strong effect [56].

The Game-of-Dice Task showed a significant difference in risky decision making between the patient and control groups (see Fig. 1), with a significantly higher number of high-risk choices in the PD group (mean patients: 10.2, SD 3.93; mean controls: 7.05, SD 3.62; Wilcoxon test; *z* = −2.596, *P* = 0.009; two-sided test, *n* = 20 in each group). The calculated effect size was *r* = 0.58, which is considered a strong effect [56].

Gender was not a significant predictor of trust behavior in a linear regression model (corrected *R*^*2*^ = 0.388; *β* = 0.180; *P* = 0.210), neither were LEDD, which includes L-DOPA and dopamine agonists (*β* = −0.151; *P* = 0.411), nor disease duration (*β* = −0.302; *P* = 0.765). Moreover, age and UPDRS III scores came close to being predictors of trust behavior, but did not reach the level of significance (age: *β* = −0.238; *P* = 0.078; UPDRS III: *β* = 0.456; *P* = 0.066). The calculated effect size for our regression study (Cohen’s f^2^) was *f*^*2*^ = 0.635 [56].

## Discussion

The main goal of this study was to empirically test whether there is a significant difference in trust behavior between non-demented PD patients without evidence of significant neuropsychiatric comorbidities (e.g., impulse control disorder, depression, or apathy) and healthy controls (matched for age, gender, education, and income). Using the trust game, an established experimental paradigm for studying trust behavior [40], we found that PD patients exhibit significantly lower levels of trust than do healthy controls. A direct comparison of the results illustrated in Fig. 1 - trust behavior (left side) versus risky decision making (right side) - shows the diametric oppositeness of trust and risk in our participants. While PD patients exhibit relatively low levels of trust behavior and relatively high levels of risk-taking behavior, healthy controls exhibit the opposite pattern. Low trust can therefore not be explained by lower levels of risky decision making in PD, measured with the Game-of-Dice Task, which is in line with previous evidence [48]. In our statistical analyses disease duration, medication, and severity of PD motor symptoms did not reach significance in predicting trust behavior.

We see these results as the starting point for future studies focusing on trust in PD patients. Our data provide an empirical basis for replication, and for theorizing in order to derive explanations for the trust deficit observed in PD patients.

As a consequence of our recruitment design (screening and excluding patients with psychiatric comorbidities, as well as playing “one-shot” trust games) we can most likely rule out the possibility that PD patients’ lower trust behavior is a secondary phenomenon of these co-morbidities, particularly dementia, impulsive-compulsive disorders, or apathy. Our statistical analyses further show that dopaminergic medication had no impact on trust behavior of PD patients in the trust game, but there was a trend for age and severity of PD motor symptoms under treatment. We cannot rule out that these variables might have reached significance in a larger sample.

Trust is based on several other constructs, particularly risk taking, reward processing and anticipation, and mentalizing ([14–16], see Background section). Our study shows that risky decision-making does not explain lower trust in PD. However, impairments in the reward and/or theory of mind systems known in PD could be possible candidates responsible for the altered trust behavior of PD patients. Independent of the question of lower trust being a primary or secondary phenomenon, this is the first report showing lower trust behavior in patients suffering from PD.

The experimental design of our study entails certain limitations. First, we acknowledge the fact that the sample size of our study, even though similar to other studies in the field (e.g. [43]), might be considered as relatively small by some readers. However, statistical power (significance) in hypotheses testing is not only influenced by sample size, but is also dependent on the effect size. Thus, our statistically significant results based on a small sample size imply a large effect size.

Second, even though our behavioral study cannot reveal the exact pathophysiology underlying the relatively low trust behavior of PD patients, it nevertheless offers potential for theorizing on possible mechanisms. PD affects virtually all brain regions known to be activated in trust situations ([19–25], see Background section). Thus, it can be hypothesized that lower trust in PD patients has a functional-anatomical basis. Obviously, further research is needed to shed light on the exact mechanisms underlying PD patients’ lowered trust behavior. This is important for a possible development of effective therapeutic interventions.

Third, we acknowledge the fact that the trust game measures trust in an economic context reflecting a significant proportion of everyday behavior, but results may not necessarily generalize to other trust situations.

Fourth, the faces used in our experimental paradigm had an age range that was lower than the mean age of our participants. Therefore, it cannot be ruled out that Parkinson patients do not show a trust deficit in social interaction with other age groups.

Fifth, even though not uncommon in neuropsychological research of PD (e.g. [43]), we cannot rule out an effect of the recruitment of spouses and family members of patients as controls on our results.

Moreover, our finding showing that medication and UPRDS III scores are not predictors of trust behavior need further empirical validation, ideally based on larger sample sizes and experimental designs including a comparison of trust behavior in off- versus on-periods and between mildly and severely affected PD patients. Importantly, even if dopaminergic medications were responsible for the trust deficit, the findings of this study would still have relevant implications for PD patients, because PD patients usually experience trust situations in their everyday life on this medication.

Another fruitful avenue for future research is the investigation of PD patients’ trustworthiness. Our study focused on the investor’s role in the trust game, thereby measuring trust. How PD patients would act in the role of the trustee (i.e., the party who is given trust and subsequently has to reciprocate, or not) is not known. Thus, it would be rewarding to study PD patients’ trustworthiness in order to find possible differences in comparison to healthy controls.

## Conclusion

In conclusion, we here show that PD patients exhibit lower levels of trust as compared to healthy controls. Now that this has been evidenced on a behavioral level, further research is needed to explore whether the trust deficit in PD patients is caused by functional impairment of relevant brain structures, medication and/or other pathophysiological conditions. Given that insights into the neurobiology of PD patients’ trust behavior are the precondition for the development of effective therapeutic interventions, future studies can be expected to provide rewarding insights.

## Footnotes

1 Income classes – 1: 0-5,000 Euros per year, 2: 5,001–10,000 Euros per year, 3: 10,001–25,000 Euros per year, 4: 25,001–50,000 Euros per year, 5: 50,001–100,000 Euros per year, 6: 100,001–200,000 Euros per year, 7: over 200,000 Euros per year.

2 Education – number of years of education starting from school entry at the age of 6 years, e.g. 12 years meaning high school graduation.
